# Elevated Carcinoembryonic Antigen at the Time of Recurrence as a Poor Prognostic Factor in Colorectal Cancer: A Propensity Score Matching Analysis

**DOI:** 10.3389/fonc.2022.821986

**Published:** 2022-06-07

**Authors:** Jung Kyong Shin, Jung Wook Huh, Woo Yong Lee, Seong Hyeon Yun, Hee Cheol Kim, Yong Beom Cho, Yoon Ah Park

**Affiliations:** Department of Surgery, Samsung Medical Center, Sungkyunkwan University School of Medicine, Seoul, South Korea

**Keywords:** carcinoembryonic antigen, recurrence, prognostic factor, colorectal cancer, adjuvant treatment

## Abstract

There are few studies on the prognostic impact of CEA level at the time of recurrence in recurrent colorectal cancer. The objective of this study was to evaluate the prognostic value of serum CEA levels at the time of recurrence in patients with recurrent colorectal cancer. Between 2007 and 2014, 962 consecutive recurrent patients for colorectal cancer were analyzed. These patients were divided into two groups according to CEA level at the time of recurrence (r-CEA): high r-CEA (≥5 ng/ml) (n = 428) and normal r-CEA (<5 ng/ml) (n = 534). The prognostic effects of r-CEA were evaluated by one-to-one propensity score matching (PSM) to adjust factors between groups. After matching, a total of 778 patients, 389 per group, were analyzed. After matching, the 5-year disease-free survival rate for the high r-CEA group was significantly lower than that for the normal r-CEA group. The 5-year overall survival rate was 56.5% in the high r-CEA group and 66.0% in the normal r-CEA group (p = 0.008). The 5-year cancer-specific survival rate was 61.7% in the high group and 67.5% in the normal group (p = 0.035). In a multivariate analysis of prognostic factors, high preoperative CEA level at the time of recurrence, poor histologic grade, and lymphatic invasion were associated with poorer overall survival. The high r-CEA level group showed significantly poorer prognosis than the normal r-CEA group. Therefore, the r-CEA level can be used as a prognostic factor in recurrent colorectal cancer. Aggressive adjuvant treatment needs to be considered for patients with an initially high CEA level and lymph node positivity who are prone to recurrence.

## Introduction

Serum carcinoembryonic antigen (CEA) measurement is a relatively simple test and has been used for tumor markers in screening and detecting recurrence in colorectal cancer patients (1–4). CEA was first known in 1965 as an antigen present in colon adenocarcinoma (5). This is one of the immunoglobulin families expressed in mucosa cells with functions such as cell recognition or adhesion (6). In colorectal cancer patients, normal cell structures are destroyed and serum CEA levels can be increased by inducing tumor cells to express CEA throughout the cell surface (7).

Locker et al. recommended CEA measurement as one of the tests to determine the treatment plan pre- and postoperative surgery (8).. Although CEA does not detect all recurrent patients, it is known as one of the effective tests for suspected recurrence in colorectal cancer patients under follow-up after radical resection (1, 9). However, serum CEA tests are performed periodically during the postoperative follow-up period and used as a test of suspected recurrence at elevated levels; not many studies have been conducted on the prognostic impact of CEA level on recurrence. Thus, the objective of this study was to investigate the prognostic impact of elevated CEA level at the time of recurrence on survival in patients with recurrent stage I–III colorectal cancer who underwent curative surgery.

## Patients and Methods

Between January 2007 and December 2014, 962 colorectal cancer patients who had recurrence after curative intent surgery at one single center were enrolled. Their clinical and pathologic characteristics were analyzed. Initial stages of patients were stage I–III colorectal cancer. This study only included colon cancer and rectal cancer patients who did not receive neoadjuvant treatment. We collected patient data from the colorectal cancer database in our institution. This study was approved by the Institutional Review Board (IRB) of Samsung Medical Center (IRB No. 2019-10-097-001). Since it was a retrospective study through medical charts, the need for written informed consent was waived by the IRB of Samsung Medical Center, Sungkyunkwan University School of Medicine.

**Figure 1:**
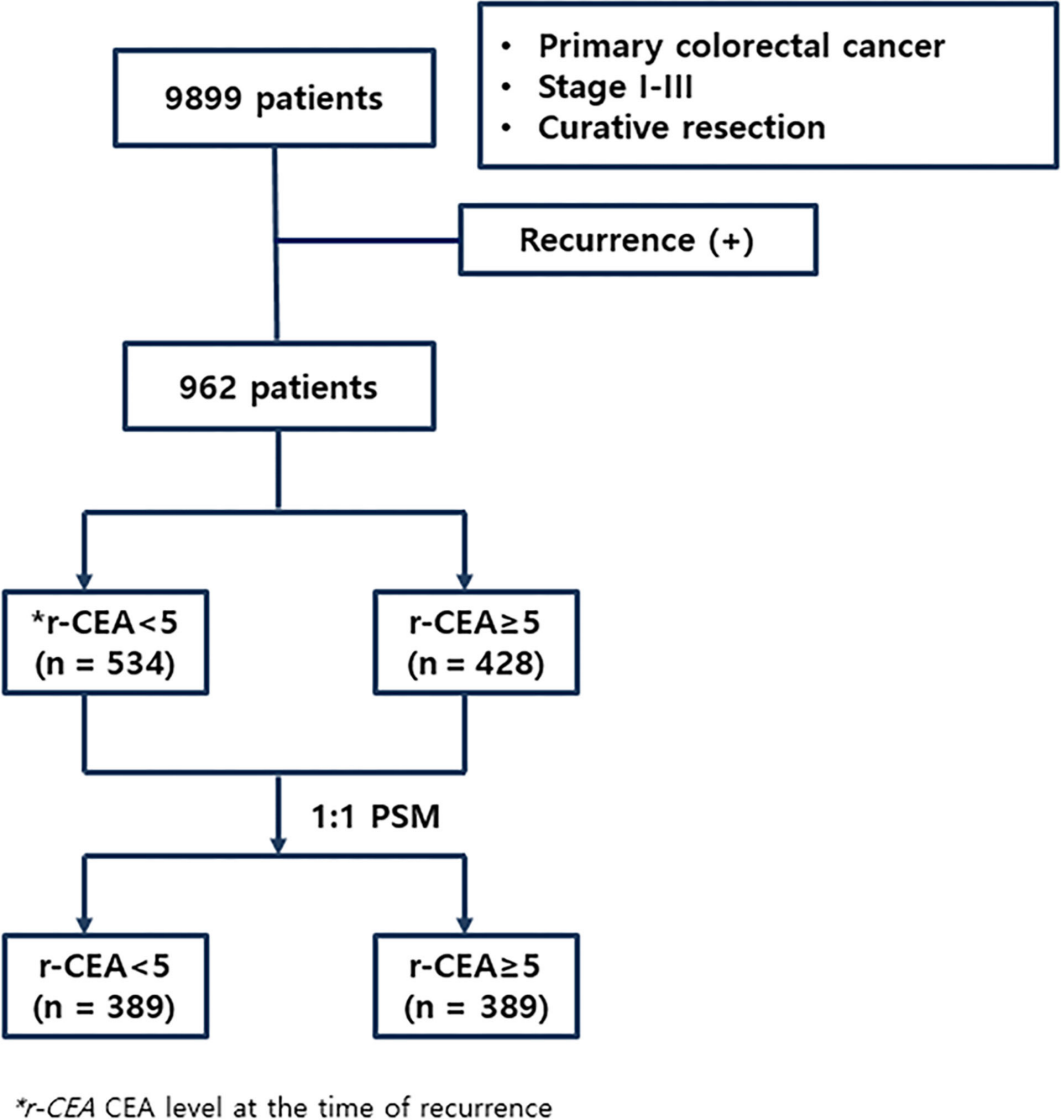
Flowchart of this study.

A serum CEA assay was performed with an automated immunochemistry analyzer (Abbott AxSYM, Abbott Laboratories, North Chicago, IL, USA) using a microparticle enzyme immunoassay with a normal range ≤ 5.0 ng/ml. Serum levels of CEA were measured preoperatively and at the time of recurrence, with CEA ≥ 5.0 ng/ml regarded as elevated. Patients were divided into two groups: 1) normal CEA at the time of recurrence (n = 534, 55.5%) and 2) high CEA at the time of recurrence (n = 428, 44.5%). **Figure 1** shows the flowchart of this study.

The definition of survival rate is as follows: OS (overall survival); survival rate after a curative surgery, DFS (disease-free survival); survival rate without recurrence after curative surgery, and LRFS (local recurrence-free survival); survival rate without local recurrence after curative surgery. During the follow-up period, laboratory tests including tumor marker determination were performed every 3 months for the first 2 years after surgery. Chest and abdominopelvic CT scans were performed every 6 months. After that, up to 5 years after surgery, tests for tumor markers and CT scans were performed every 6 months. Endoscopy was performed at 1, 3, and 5 years after surgery. If there were any elevated findings at the time of performing tests only for tumor markers, an additional image test was performed. If necessary, a PET CT scan was performed. Tumor markers and image tests such as CT scans were synthesized to determine whether there was a recurrence. The recurrence date was defined as the day when clinicians confirmed the recurrence according to the imaging or pathological results and described in the chart. Decisions on treatment such as surgery, chemotherapy, or radiotherapy after recurrence were discussed in a multidisciplinary team that included surgeons, medical oncologists, radiologists, and other related professions.

### Statistical Analysis

We performed statistical analyses using SPSS for Windows version 26.0 (SPSS, Chicago, IL, USA). The chi-squared test, Fisher’s exact test, and Mann–Whitney U test were used to analyze the differences between the two groups. The oncologic effects of serum CEA were evaluated by one-to-one propensity score matching to adjust factors, including age, sex, preoperative CEA level, tumor location and size, cell differentiation, pathologic T and N stage, lymphatic/perineural/vascular invasion, and tumor budding. Survival rates were calculated through the Kaplan–Meier method and log-rank test. Multivariate analysis was performed using the Cox proportional hazard model. When *p* was less than 0.05, it was interpreted as a statistically meaningful result.

## Results

### Clinicopathologic Characteristics of Patients Before and After Propensity Score Matching

Among all patients with recurrence, 534 (55.5%) patients had a normal CEA level at the time of recurrence (r-CEA) and 428 (44.5%) presented a high r-CEA level. As shown in **Table 1**, many variables were differently distributed between patients with normal and high r-CEA levels before PSM. Of the 428 patients with high r-CEA levels, 149 (34.8%) also had high preoperative serum CEA levels (p < 0.001). Patients with high r-CEA levels were more likely to have aggressive initial histologic features. Advanced stage, poor histology, presence of lymphatic/perineural invasion, positive tumor budding, and the rate of receiving adjuvant treatment were more common among patients with high r-CEA levels than in those with normal r-CEA levels. The median disease-free interval was significantly shorter in the high r-CEA group (19.8 vs. 17.7 months, p = 0.028). The treatment modality after recurrence was similar between the two groups.

**Table 1:** Patient clinicopathologic characteristics before and after propensity score matching analysis.

	Before propensity score matching			After propensity score matching		
	r-CEA <5 (n = 534)	r-CEA ≥5 (n = 428)	*p*	r-CEA <5 (n = 389)	r-CEA ≥5 (n = 389)	*p*
Age, years, median (SD)	60 ± 13	61 ± 13	0.680	60 ± 13	61 ± 12	0.392
Sex, n (%)			0.514			0.770
Male Female	323 (60.5) 211 (39.5)	250 (58.4) 178 (41.6)		233 (59.9) 156 (40.1)	229 (58.9) 160 (41.1)	
Initial CEA (ng/mL)			<0.001			0.203
Normal (<5) High (>5)	406 (76.0) 128 (24.0)	279 (65.2) 149 (34.8)		287 (73.8) 102 (26.2)	270 (69.4) 119 (30.6)	
Initial tumor location, n (%)			0.105			0.498
Colon Rectum	326 (61.0) 208 (39.0)	283 (66.1) 145 (33.9)		250 (64.3) 139 (35.7)	259 (66.6) 130 (33.4)	
Initial TNM stage, n (%)			<0.001			1.000
I II III	68 (12.7) 144 (27.0) 322 (60.3)	14 (3.3) 71 (16.6) 343 (80.1)		14 (3.6) 70 (18.0) 305 (78.4)	14 (3.6) 70 (18.0) 305 (78.4)	
Initial pathologic T stage, n (%)			<0.001			0.610
T1 T2 T3 T4	41 (7.7) 46 (8.6) 303 (56.7) 144 (27.0)	12 (2.8) 17 (4.0) 279 (65.2) 120 (28.0)		10 (2.6) 22 (5.7) 239 (61.4) 118 (30.3)	10 (2.6) 15 (3.9) 252 (64.8) 112 (28.7)	
Initial pathologic N stage, n (%)			<0.001			0.672
N0 N1 N2	212 (39.7) 147 (27.5) 175 (32.8)	85 (19.9) 164 (38.3) 179 (41.8)		84 (21.6) 138 (35.5) 167 (42.9)	84 (21.6) 149 (38.3) 156 (40.1)	
Initial size of tumor (cm, SD)	4.7 ± 3.0	5.1 ± 2.2	0.076	5.0 ± 3.1	5.1 ± 2.2	0.784
Initial cell differentiation, n (%)			0.293			0.922
WD+MD PD+MUC+SRC	462 (86.5) 72 (13.5)	360 (84.1) 68 (15.9)		327 (84.1) 62 (15.9)	328 (84.3) 61 (15.7)	
Initial lymphatic invasion, n (%)			0.004			0.565
(+) (-)	245 (45.9) 289 (54.1)	236 (55.1) 192 (44.9)		213 (54.8) 176 (45.2)	205 (52.7) 184 (47.3)	
Initial venous invasion, n (%)			0.422			0.393
(+) (-)	152 (28.5) 382 (71.5)	132 (30.8) 296 (69.2)		125 (32.1) 264 (67.9)	114 (29.3) 275 (70.7)	
Initial perineural invasion, n (%)			0.042			0.705
(+) (-)	158 (29.6) 376 (70.4)	153 (35.7) 275 (64.3)		135 (34.7) 254 (65.3)	130 (33.4) 259 (66.6)	
Initial tumor budding, n (%)			0.048			0.828
(+) (-)	274 (51.3) 260 (48.7)	247 (57.7) 181 (42.3)		222 (57.1) 167 (42.9)	219 (56.3) 170 (43.7)	
MSI			0.698			0.521
MSS MSI-H MSI-L	505 (94.6) 21 (3.9) 8 (1.5)	402 (93.9) 21 (4.9) 5 (1.2)		372 (95.6) 13 (3.3) 4 (1.0)	365 (93.8) 19 (4.9) 5 (1.3)	
KRAS			0.360			0.963
Wild type Mutation type Not identification	385 (72.1) 88 (16.5) 61 (11.4)	311 (72.6) 79 (18.5) 38 (8.9)		276 (71.0) 74 (19.0) 39 (10.0)	279 (71.7) 73 (18.8) 37 (9.5)	
Median disease-free interval, months	19.8 ± 15.4	17.7 ± 14.4	0.027	18.7 ± 14.0	17.8 ± 14.9	0.374
Treatment after recurrence, n (%)			0.625			0.764
Salvage treatment Surgery +/- CTx, RTx CTx or RTx or CCRT only Conservative treatment	439 (82.2) 205 (38.4) 234 (43.8) 95 (17.8)	358 (83.7) 177 (41.4) 181 (42.3) 70 (16.3)		329 (84.6) 141 (36.3) 188 (48.3) 60 (15.4)	324 (83.3) 132 (33.9) 192 (49.4) 65 (16.7)	

CEA, carcinoembryonic antigen; r-CEA, CEA level at the time of recurrence; WD, well-differentiated; MD, moderately differentiated; PD, poorly differentiated; MUC, mucinous adenocarcinoma; SRC, signet ring cell carcinoma; MSS, microsatellite stable; MSI-L, microsatellite instability low; MSI-H, microsatellite instability high; CTx, chemotherapy; RTx, radiotherapy.

Based on these findings, we performed PSM with an adjusted ratio of 1:1. A total of 778 patients were matched (389 in each group). After PSM, two groups were well balanced for all variables (**Table 1**).

Following recurrence, before matching, 439 (82.2%) patients with a normal r-CEA level underwent salvage treatment while 358 (83.7%) patients with a high r-CEA level underwent salvage treatment (p = 0.625). In matched patients, 329 (84.6%) patients with a normal r-CEA level received salvage treatment and 324 (83.3%) patients with a high r-CEA level received salvage treatment (p = 0.764).

### Survival According to CEA Level at the Time of Recurrence Before and After Propensity Score Matching

**Figure 2:**
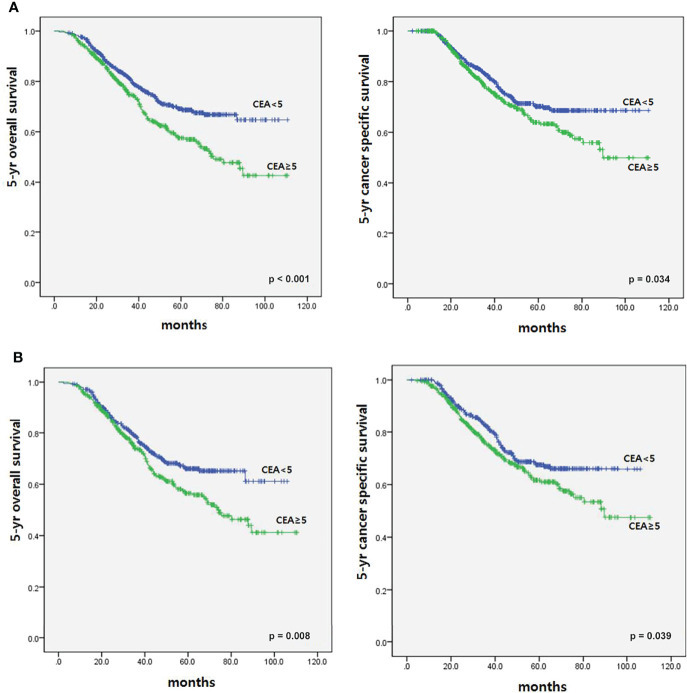
Survival according to CEA level at the time of recurrence before and after matching. **(A)** Before matching. **(B)** After matching.

**Figure 3:**
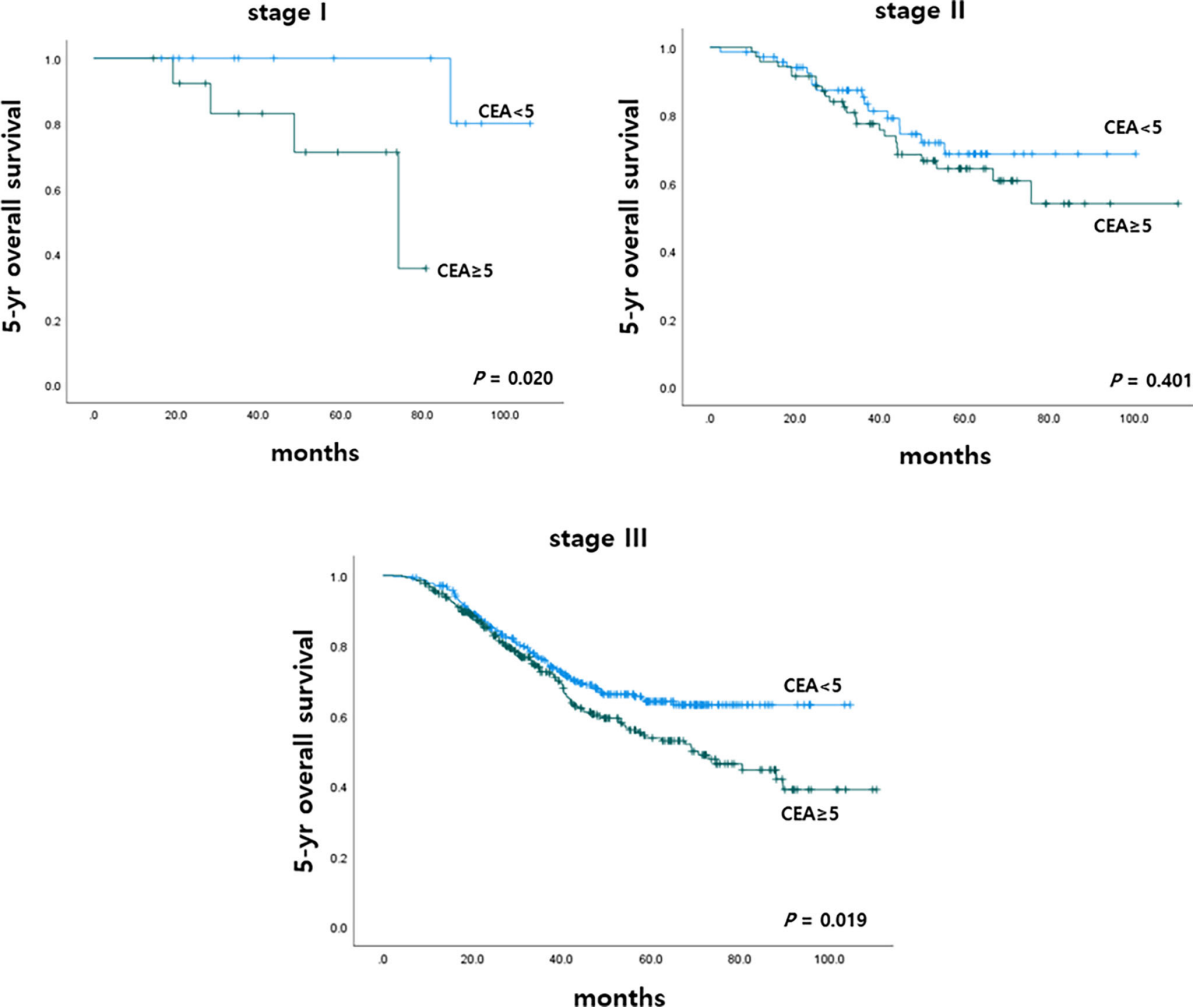
Five-year overall survival according to CEA level at the time of recurrence in matched patients.

**Figure 4:**
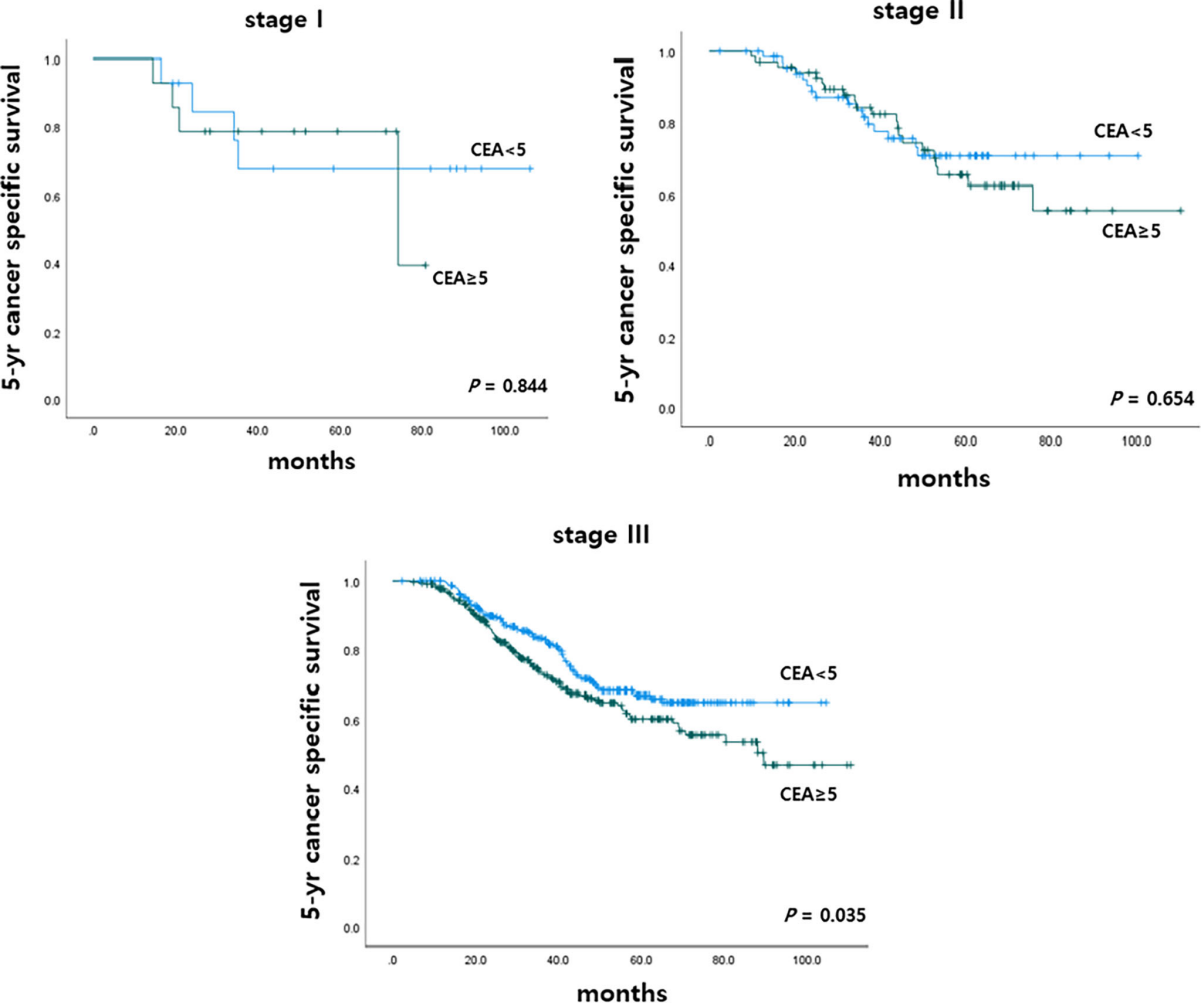
Five-year cancer specific survival according to CEA level at the time of recurrence in matched patients.

To determine the impact of r-CEA level on oncologic outcomes, we analyzed the 5-year overall survival (OS) and 5-year cancer-specific survival (CSS) rates according to r-CEA level. Before matching, patients with a high r-CEA level showed significantly lower 5-year OS (57.6% vs. 69.1%, p *<* 0.001) and 5-year CSS (63.9% vs. 70.2%, p = 0.034) than patients with a normal r-CEA level (**Figure 2A**). Analysis of matched patients showed similar results. Patients with a high r-CEA level showed significantly lower 5-year OS (56.5% vs. 66.0%, p = 0.008) and 5-year CSS (61.7% vs. 67.5%, p = 0.039) than patients with a normal r-CEA level (**Figure 2B**). **Figure 3** and **Figure 4** show the 5-year OS and CSS rates according to the cancer stage. Five-year OS rates were shown to be significantly lower in stage I (71.2% vs. 80.0%, p = 0.020) and stage III (53.7% vs. 64.1%, p = 0.019) in the patients with high r-CEA (**Figure 3**). In terms of 5-year CSS rates, only stage III was shown to be significantly lower in the high r-CEA group (60.1% vs. 66.8%, p = 0.035) (**Figure 4**).

### Survival According to Initial CEA and CEA Level at the Time of Recurrence Before and After Propensity Score Matching

**Figure 5:**
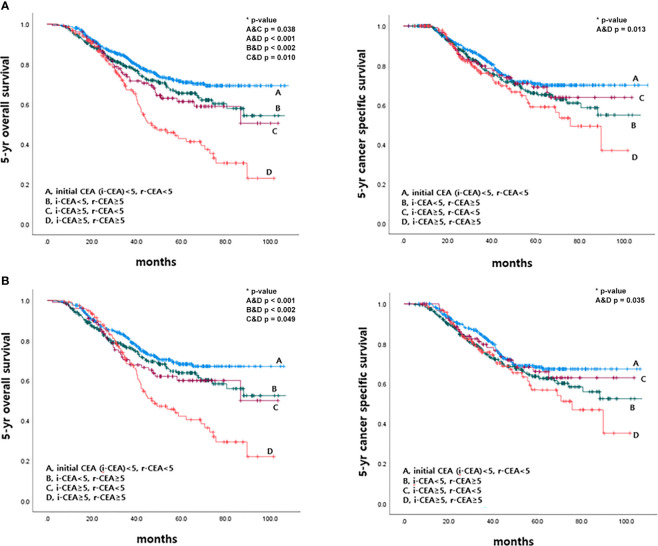
Survival according to initial and CEA level at the time of recurrence before and after matching. **(A)** Before matching. **(B)** After matching.

This study also analyzed 5-year OS and 5-year CSS rates according to initial CEA and r-CEA levels. When both initial CEA and r-CEA were high, the cancer-specific survival (CSS) rate was significantly worse than in other cases. For a case with a normal initial CEA and a high r-CEA, the prognosis was better than when both initial CEA and r-CEA were high. If both initial CEA and r-CEA were high, the survival rate was significantly worse than that in the other case when both initial CEA and r-CEA were normal (**Figure 5**).

### Survival According to CEA Level at the Time of Recurrence and Pathologic Nodal Status Before and After Propensity Score Matching

**Figure 6:**
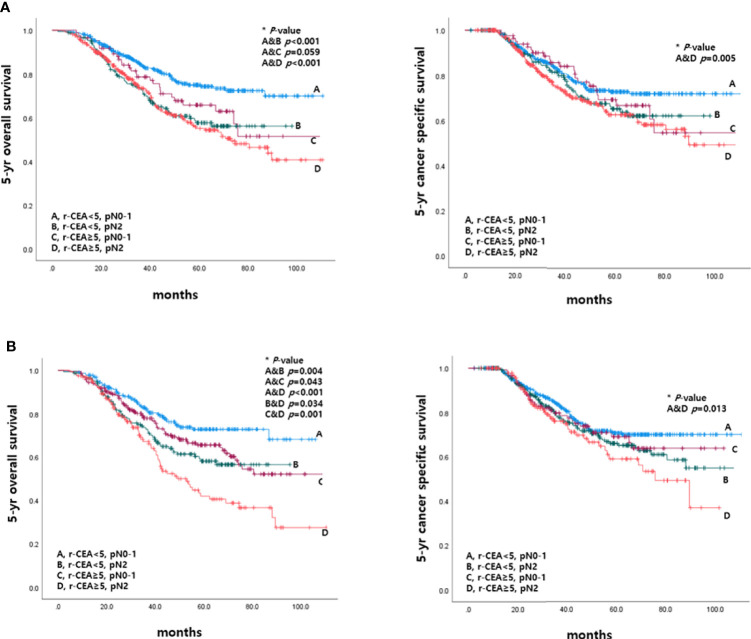
Survival according to CEA level at the time of recurrence and pathologic nodal status before and after matching. **(A)** Before matching. **(B)** After matching.

This study also analyzed 5-year OS and 5-year CSS rates according to r-CEA level and pathologic nodal status (pN). Patients with r-CEA ≥5 and pN2 had significantly lower 5-year OS and CSS rates than other patients. In the patients with r-CEA ≥5 and pN2, the survival rate was significantly worse than that in the other case when r-CEA was normal and pN0-1 (**Figure 6**).

### Prognostic Factors for OS and CSS

To determine whether r-CEA elevation is an independent prognostic factor, an analysis was performed using the Cox proportional hazard model. On univariate analysis (**Table 2**), factors associated with poorer overall survival included age ≥65 years, high initial CEA level, high r-CEA level, initial advanced T and N stage, poor histology, lymphatic invasion, disease-free interval less than 12 months, and conservative treatment after recurrence. In multivariate analysis, age, high initial and r-CEA levels, initially advanced T and N stage, poor histology, lymphatic invasion, disease-free interval less than 12 months, and conservative treatment after recurrence were associated with poorer overall survival.

**Table 2:** Prognostic factors of survival for matched patients.

Factors	Overall survival			Cancer-specific survival		
	Univariate	Multivariate		Univariate	Multivariate	
	*p*	HR (95% CI)	*p*	*p*	HR (95% CI)	*p*
Initial CEA (ng/L)						
≥5 versus <5	0.016	1.574 (1.221–2.029)	<0.001	0.553		
CEA at recurrence (ng/L)						
≥5 versus <5	0.024	1.395 (1.088–1.788)	0.009	0.019	1.322 (1.104–1.723)	0.039
Age (years)						
≥65 versus <65	<0.001	2.055 (1.604–2.633)	<0.001	0.002	1.460 (1.114–1.913)	0.006
Gender						
Female versus male	0.787			0.497		
Tumor location						
Rectum versus colon	0.772			0.378		
Initial T stages						
2 versus 1	0.498	1.789 (1.038–3.083)	0.036	0.076	0.992 (0.428–2.298)	0.985
3 versus 1	0.029	1.593 (1.018–2.494)	0.041	0.045	1.866 (0.987–3.530)	0.055
4 versus 1	0.015	2.006 (1.269–3.171)	0.003	0.007	2.526 (1.311–4.866)	0.006
Initial N stages						
1 versus 0	0.036	1.044 (0.863–1.264)	0.657	0.040	1.080 (0.748–1.559)	0.680
2 versus 0	0.028	1.290 (1.070–1.556)	0.008	0.002	1.776 (1.266–2.493)	0.001
Initial cell type						
PD/MUC/SRC versus	0.002	2.293 (1.712–3.071)	<0.001	0.014	1.801 (1.286–2.522)	0.001
WD/MD						
Initial lymphatic invasion						
Yes versus no	0.036	1.735 (1.346–2.237)	<0.001	0.092		
Initial venous invasion						
Yes versus no	0.529			0.756		
Initial perineural invasion						
Yes versus no	0.761			0.033	1.820 (1.388–2.385)	<0.001
Initial tumor budding						
Yes versus no	0.091			0.045	1.575 (1.199–2.069)	0.001
KRAS						
Mutation versus wild	0.161			0.577		
Disease-free interval						
≥12 versus <12 months	<0.001	3.200 (2.494–4.106)	<0.001	<0.001	2.242 (1.716–2.930)	<0.001
Treatment after recurrence						
Surgery versus conserve	<0.001	0.235 (0.167–0.331)	<0.001	<0.001	0.251 (0.176–0.358)	<0.001
CTx or RTx versus conservative	<0.001	0.220 (0.159–0.304)	<0.001	<0.001	0.179 (0.125–0.255)	<0.001

CEA, carcinoembryonic antigen; WD, well-differentiated; MD, moderately differentiated; PD, poorly differentiated; MUC, mucinous adenocarcinoma; SRC, signet ring cell carcinoma; CTx, chemotherapy; RTx, radiotherapy.

Results were similar for cancer-specific survival. In multivariate analysis, age, high r-CEA level, initial lymphovascular invasion, advanced T and N stage, poor histology, presence of perineural invasion and tumor budding, disease-free interval less than 12 months, and conservative treatment after recurrence were associated with poorer cancer-specific survival (**Table 2**).

### Prognostic Factors for High CEA at the Time of Recurrence

Furthermore, initially high CEA level and pathologic node positivity were independent poor prognostic factors in high r-CEA level. Univariate and multivariate analyses were performed to evaluate independent prognostic factors related to a high level of CEA at the time of recurrence. On univariate analysis, factors associated with high r-CEA level included high initial CEA level and initial advanced N stage. In multivariate analysis, high initial CEA and initial advanced N stage still showed a significant association with high r-CEA level (**Table 3**).

**Table 3:** Prognostic factors of high CEA at the time of recurrence.

Factors	CEA at recurrence ≥5		
	Univariate	Multivariate	
	*p*	HR (95% CI)	*p*
Initial CEA (ng/L)			
≥5 versus <5	0.007	1.694 (1.279–2.244)	<0.001
Gender			
Female versus male	0.354		
Tumor location			
Rectum versus colon	0.266		
Initial T stages			
2 versus 1	0.261.		
3 versus 1	0.246		
4 versus 1	0.087		
Initial N stages			
1 versus 0	0.416	2.783 (1.989–3.894)	<0.001
2 versus 0	<0.001	2.551 (1.840–3.537)	<0.001
Initial cell type			
PD/MUC/SRC versus	0.757		
WD/MD			
Initial lymphatic invasion			
Yes versus no	0.745		
Initial venous invasion			
Yes versus no	0.132		
Initial perineural invasion			
Yes versus no	0.904		
Initial tumor budding			
Yes versus no	0.772		
Disease free interval			
≥12 versus <12 months	0.059	0.773 (0.593–1.006)	0.056

CEA, carcinoembryonic antigen; WD, well-differentiated; MD, moderately differentiated; PD, poorly differentiated; MUC, mucinous adenocarcinoma; SRC, signet ring cell carcinoma.

### Patterns of Recurrence According to CEA Level at the Time of Recurrence After Propensity Score Matching

Regarding the recurrence site, there were no differences between the two groups in terms of locoregional and distant metastasis. The characteristics of these patients are summarized in **Table 4**.

**Table 4:** Long-term oncologic outcomes of matched cohorts.

	r-CEA <5 (n = 389)	r-CEA ≥5 (n = 389)	*p*
Recurrence site			0.034
Locoregional	49 (12.6)	45 (11.6)	0.742
Liver	126 (32.4)	121 (31.1)	0.758
Lung	119 (30.6)	119 (30.6)	1.000
Peritoneal seeding	19 (4.9)	34 (8.7)	0.033
Distant lymph node	29 (7.5)	54 (13.9)	0.004
Other (ovary, brain, bone)	32 (8.2)	31 (7.9)	0.895

r-CEA, CEA level at the time of recurrence.

## Discussion

To the best of our knowledge, this is the first study to evaluate the prognostic significance of r-CEA level using propensity score matching for recurrent colorectal cancer patients. In this study, patients with recurrent colorectal cancer who had an elevated CEA level at the time of recurrence showed poorer 5-year OS and CSS than those with a normal CEA level. Our results also revealed that elevated r-CEA level was an independent factor for poor prognosis in matched groups. These results supported that, in addition to other oncological factors, r-CEA elevation was also an independent risk factor of poor survival outcomes, indicating the necessity of using more aggressive adjuvant treatment for patients with recurrent colorectal cancer.

Serial examination of CEA after colorectal cancer surgery is generally recommended. The sensitivity to recurrence detection has been reported to be 70%–80% (3, 10, 11). Several studies have reported that preoperative CEA elevation (>5 ng/ml) or up to two times the normal cutoff value has significantly decreased survival outcomes (12–16). However, evidence is limited regarding the prognostic impact of CEA level at the time of recurrence on patients with recurrent colorectal cancer.

This current study demonstrated that high r-CEA level was a significant prognostic factor associated with poor 5-year OS and CSS in patients with recurrent colorectal cancer. Moreover, this study did propensity score matching for survival analysis to overcome the confounding bias of patient characteristic differences between groups. As a result, high r-CEA level was identified as an independent poor prognostic factor even after adjusting for confounding factors. This study also showed that the initial N2 stage is one of the prognostic factors of survival and high r-CEA. Based on this, it was suggested that more aggressive treatment be considered, as the initial N2 stage is likely to coincide with a high r-CEA, and in this case, the prognosis is likely to be poor.

It was not clear why patients with higher CEA levels at the time of recurrence had poor oncological results. Several studies have suggested why high CEA levels show poor prognosis (17, 18). Jessup et al. (17) have demonstrated that tumor cells that produce CEA have higher tumorigenic potential and ability to spread distantly. Such a result might be facilitated by the role of CEA in cell adhesion. Scurr et al. (18) have suggested that an adoptive immune response of CEA-specific T cells causes enteropathy, increasing epithelial leakage while losing mucosal integrity, thereby promoting tumor growth or recurrence. Whatever the reason, patients with a high r-CEA level are expected to show poor oncologic outcomes. Although r-CEA alone is not sufficient to predict expected survival, this study is meaningful in suggesting that r-CEA is one of the important factors to predict the survival of recurrent colorectal cancer patients.

Recently, several studies have been reported to predict recurrence and prognosis through biomarkers such as circulating tumor DNA (ctDNA) and microsatellite installation (MSI) status (19, 20). In our institution, ctDNA testing is not yet performed as a routine. According to previous studies, clinical application using them is considered meaningful. It is thought to be used with r-CEA level to help predict the prognosis of recurrent patients and to determine the treatment modality. We are planning a study on this and will report the results later.

This study had some limitations, including its retrospective nature in a single center. In addition, we did not consider benign conditions such as heavy smokers and liver disease that might elevate serum CEA levels. Furthermore, this study did not analyze changes in CEA levels during the course of the disease. Despite the limitations, this study is meaningful in that it has shown that the prognosis after recurrence can be predicted through the measurement of CEA, a relatively easy test that has been serially examined after curative surgery in colorectal cancer. This study is expected to be of clinical value, such as helping to predict prognosis of recurrent patients and determine treatment modality through CEA, a test performed serially after surgery in clinical practice.

In conclusion, elevation of the CEA level at the time of recurrence is an independent prognostic factor for survival outcome of patients with colorectal cancer after curative intent surgery. Therefore, this study showed that serial monitoring of serum CEA after curative surgery is an important factor in predicting the prognosis after recurrence of colorectal cancer as well as suspected recurrence. Selection of aggressive treatment strategies based on r-CEA level might improve patient outcomes. Furthermore, initially high CEA level and pathologic node positivity are independent poor prognostic factors in high r-CEA level patients. In light of this evidence, aggressive adjuvant treatment can be considered in patients with the above factors who are prone to recurrence. In the future, a multi-institutional prospective study should also be conducted.

## Data Availability Statement

The original contributions presented in the study are included in the article/**Supplementary Material**. Further inquiries can be directed to the corresponding author.

## Ethics Statement

Written informed consent was obtained from the individual(s) for the publication of any potentially identifiable images or data included in this article.

## Author Contributions

Study design: JS, JH; data acquisition: JS, JH, YP; data analysis and interpretation: JS, JH, HK; manuscript preparation: JS, JH, YC; manuscript editing: JS, JH, SY; manuscript review: JS, JH, WL. All authors contributed to the article and approved the submitted version.

## Conflict of Interest

The authors declare that the research was conducted in the absence of any commercial or financial relationships that could be construed as a potential conflict of interest.

## Publisher’s Note

All claims expressed in this article are solely those of the authors and do not necessarily represent those of their affiliated organizations, or those of the publisher, the editors and the reviewers. Any product that may be evaluated in this article, or claim that may be made by its manufacturer, is not guaranteed or endorsed by the publisher.
