# Supplementary material for: Elevated Carcinoembryonic Antigen at the Time of Recurrence as a Poor Prognostic Factor in Colorectal Cancer: A Propensity Score Matching Analysis
Source: Front Oncol. 2022 Jun 7;12:821986. doi: 10.3389/fonc.2022.821986 (PMC9209715; doi:10.3389/fonc.2022.821986)
Supplement: Supplementary file 1 [file DataSheet_1.docx]

**Supplementary Data**

Supplementary figure 1 and 2 are examples of the longitudinal values of CEA levels during the follow up period. In this result, two patients who died of poor prognosis after recurrence were described.

Supplementary Figure 1 shows the data of a patient who has undergone surgery for sigmoid colon cancer. The preoperative CEA level was less than 5, and the postoperative CEA level maintained well below 5. During follow-up, the CEA level rose to 6.75, and lung metastasis was confirmed by chest CT.
 Supplementary Figure 2 shows the data of a patient who underwent surgery for rectal cancer. The preoperative CEA level was 14.8, which was quite high. It decreased to 2.7 after surgery and maintained below 5. During follow-up, the CEA level rose to more than 10 and was diagnosed as lung metastasis. Since then, the patient has continued to receive chemotherapy, but has died due to poor prognosis.

**Figure Legends**

**Supplementary Figure 1 |** Longitudinal value of CEA

**Supplementary Figure 2 |** Longitudinal value of CEA
