# Supplementary figures and images for: Elevated Carcinoembryonic Antigen at the Time of Recurrence as a Poor Prognostic Factor in Colorectal Cancer: A Propensity Score Matching Analysis
Source: Front Oncol. 2022 Jun 7;12:821986. doi: 10.3389/fonc.2022.821986 (PMC9209715; doi:10.3389/fonc.2022.821986)

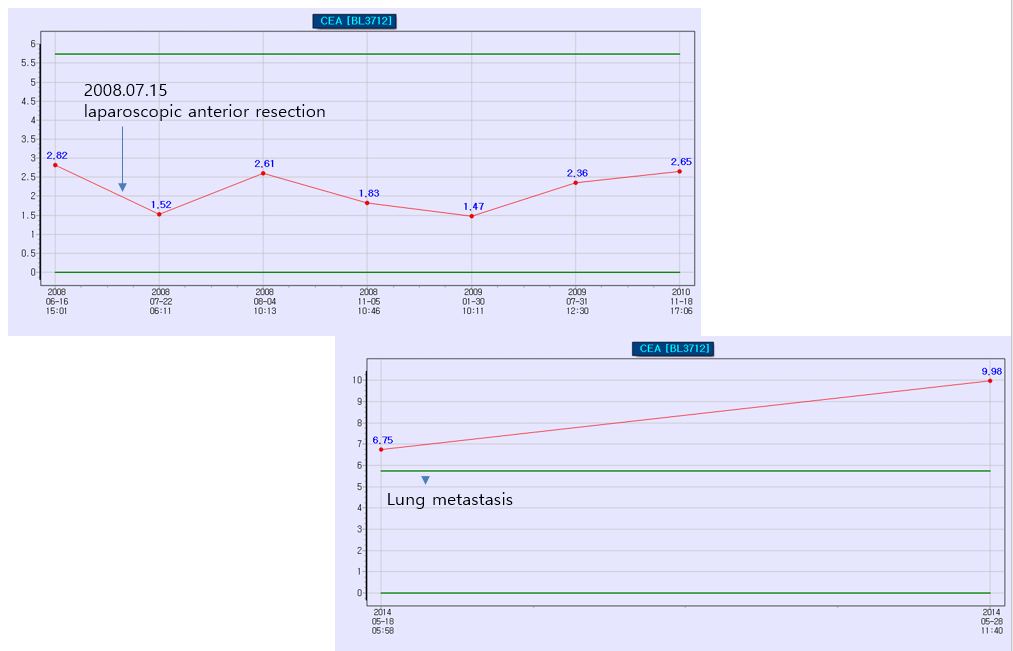

Supplement: Supplementary file 2 [file Image_1.tif]

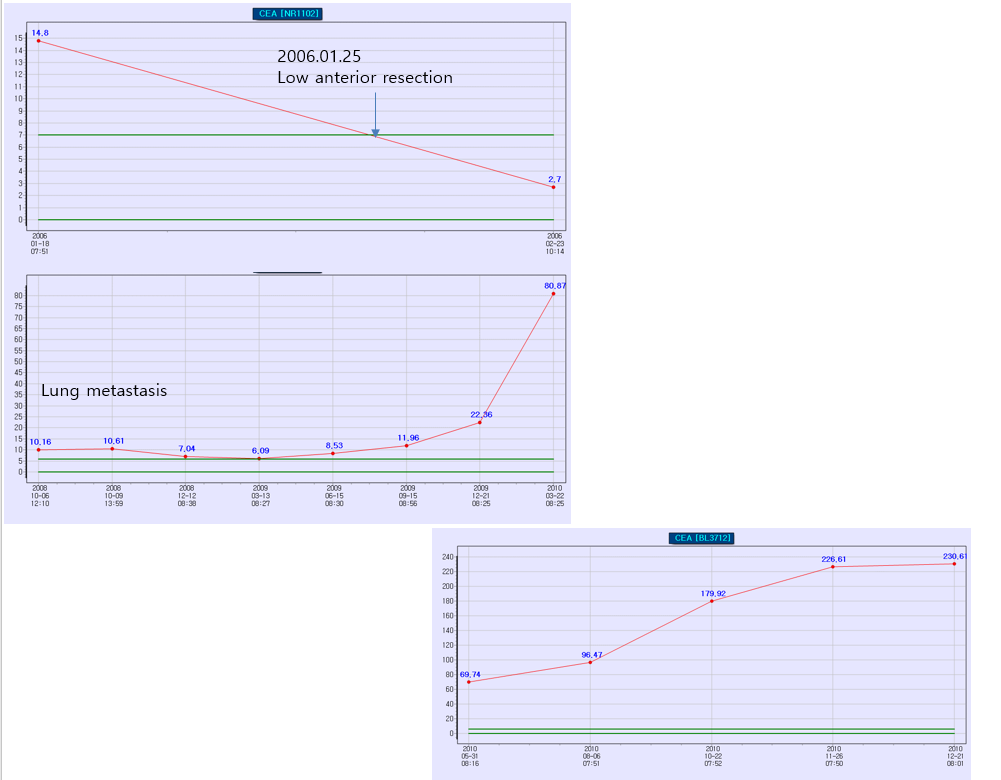

Supplement: Supplementary file 3 [file Image_2.tif]
